# Radiation resistance in head and neck squamous cell carcinoma: dire need for an appropriate sensitizer

**DOI:** 10.1038/s41388-020-1250-3

**Published:** 2020-03-10

**Authors:** Marsha-Kay N. D. Hutchinson, Michelle Mierzwa, Nisha J. D’Silva

**Affiliations:** 1 Department of Periodontics and Oral Medicine, University of Michigan School of Dentistry, 1011 North University Ave, Ann Arbor, MI 48109-1078 USA; 2 Department of Radiation Oncology, University of Michigan Medical School, Ann Arbor, MI USA; 3 Department of Pathology, University of Michigan Medical School, Ann Arbor, MI USA

**Keywords:** Oral cancer, Cancer therapeutic resistance

## Abstract

Radiation is a significant treatment for patients with head and neck cancer. Despite advances to improve treatment, many tumors acquire radiation resistance resulting in poor survival. Radiation kills cancer cells by inducing DNA double-strand breaks. Therefore, radiation resistance is enhanced by efficient repair of damaged DNA. Head and neck cancers overexpress EGFR and have a high frequency of p53 mutations, both of which enhance DNA repair. This review discusses the clinical criteria for radiation resistance in patients with head and neck cancer and summarizes how cancer cells evade radiation-mediated apoptosis by p53- and epidermal growth factor receptor (EGFR)-mediated DNA repair. In addition, we explore the role of cancer stem cells in promoting radiation resistance, and how the abscopal effect provides rationale for combination strategies with immunotherapy.

## Introduction

**Fig. 1:**
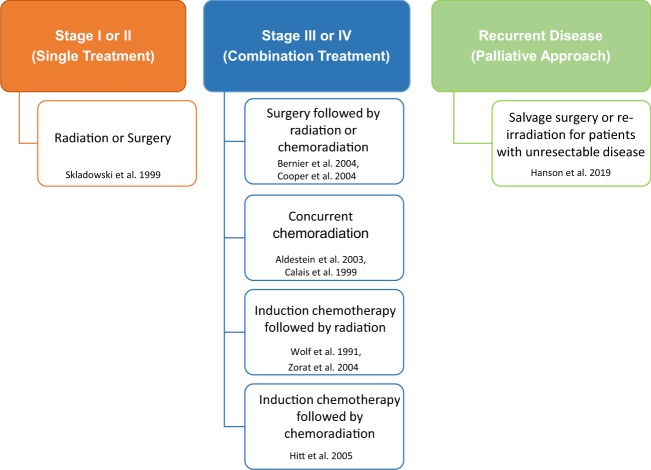
Role of radiation in treatment of HNSCC based on stage. Early stage disease is treated with a unimodality approach while later stage disease requires a multimodality approach.

Globally, over 650,000 head and neck cancers are diagnosed each year, and ~50% of patients succumb to their disease [1]. More than 90% of head and neck cancers are squamous cell carcinomas (HNSCC). In the United States, HNSCC account for 4% of all cancers. It is projected that in 2020 alone over 53,000 people in the United States will develop the disease and nearly 11,000 people will die within the year [2]. Some of the major risk factors include tobacco use, alcohol consumption, and human papilloma virus 16 (HPV16) infection. Treatment decisions are determined by taking into consideration factors such as the primary site, stage, surgical accessibility, associated morbidity, and the patient’s general health. Radiation is one of the chief treatment modalities for the management of HNSCC. Early stage disease is typically treated with radiation or surgery; locoregionally advanced disease is usually tackled with combined approaches including surgery followed by adjuvant therapy, definitive chemoradiation, or bioselection [3]. Therefore, a critical determinant of local control of HNSCC is dependent on the tumor’s sensitivity to radiation (Fig. 1). Overexpression of epidermal growth factor receptor (EGFR) and p53 mutations have been linked to treatment resistance in HNSCC. EGFR is overexpressed in 90% of HNSCCs and p53 is the most common somatic mutation [4]. Both EGFR and p53 are implicated in repairing radiation-induced DNA damage. This review discusses the clinical criteria for radiation resistance in patients with HNSCC and summarizes how cancer cells evade radiation-mediated apoptosis by p53- and EGFR-mediated DNA repair. Additional entities such as the presence of cancer stem cells (CSCs) as a cause of resistance are also addressed.

## Radiation resistance

The significant role of ionizing radiation for the management of HNSCC is irrefutable. Currently, a large number of patients with HNSCC undergo some form of external beam radiotherapy (EBRT). Intensity-modulated radiation therapy (IMRT) is a form of EBRT that delivers very precise radiation doses to the entire tumor or specific areas within the tumor, utilizing computer-controlled X-ray accelerators that conform to the three-dimensional structure of the tumor. This helps to significantly reduce damage to neighboring normal tissue during treatment. Alternatively, radiation may be internally administered utilizing radioactive implants (brachytherapy) that are removed when the appropriate dose has been administered.

Proton therapy is a recent advance in radiotherapy. Bragg peak refers to the physical properties of a proton beam that allow rapid decrease in radiation dose beyond the tumor, leading to lower normal tissue doses. Phase II data demonstrated efficacy, safety, and favorable toxicity profiles in base of skull malignancies, periorbital tumors, nasopharynx cancer, and re-irradiation while studies in other head and neck malignancies are ongoing [5, 6]. Furthermore, the Patel et al.’s meta-analysis of multiple single institution series has shown very favorable 5-year overall survival of patients treated with charged particles compared with those treated with photon radiotherapy [7]. However, there is not yet Phase III data in HNSCC to suggest improved tumor control or decreased toxicity associated with the use of proton-beam therapy. NCT01893307 is an ongoing Phase II/III randomized trial in oropharyngeal cancer that compares outcomes after chemoradiation given by proton versus photon therapy. The results from this trial should provide insight after its completion.

Patients with HNSCC typically receive ionizing radiation over several weeks in daily sessions on weekdays. A standard dose of 70 Gy in 35 fractions is usually given over 7 weeks. The doses may be administered via accelerated fractionation, hyperfractionation, or hypofractionation. Accelerated fractionation involves dividing the total dose into small doses that may be given more than once per day with reduction in total treatment time. Accelerated fractionation and particularly hyperfractionation (twice daily treatment) are more efficacious in maintaining local tumor control and improving disease-free survival over standard fractionation radiation alone, with inferior outcomes compared with concurrent chemoradiation [8]. Hypofractionation involves administering larger doses once per day or less frequently but spans a shorter period than the standard therapy. Hypofractionation is not commonly used in the definitive management of HNSCC due to concerns of late side effects including dysphagia and fibrosis.

Locoregional relapse is a significant cause of patient mortality and morbidity, and is an indicator of treatment failure [9]. Within 5 years from the end of treatment, approximately a third of patients with HNSCC develop locoregional failure and their prognosis is significantly attenuated [10]. p16-negative disease is a risk factor for locoregional failure following definitive treatment [11]. Locoregional control is also critically important to quality of life as tumor progression can lead to deficits in speech, eating, social interactions, physical deformities, and painful nonhealing wounds.

Although radiation eradicates a large fraction of tumor cells, selected groups of tumor cells (clonogens) are able to survive and repopulate irradiated areas. While 5-year overall survival ranges from 40 to 60% in patients treated exclusively with radiation or chemoradiation, ~25% of patients treated with radiation suffer from local relapse [12]. Tumors are labeled as “radiation-resistant” if recurrences are observed within 6 months following the first course of radiation. Treatment options are significantly diminished for patients with recurrent disease or second primary tumors in the previously irradiated field. The time a patient remains disease-free prior to tumor recurrence is a significant factor when deciding the therapeutic benefit of re-irradiation. Surgical salvage is often the primary treatment modality following failure of radiation [13] with reported 5-year survival rates up to 30%. Patients with positive margins, lymphovascular invasion, perineural invasion, and extra nodal extension are deemed high risk for recurrence subsequent to surgical salvage. In addition, some patients may benefit from post-operative re-irradiation, which was reported to improve progression-free survival in combination with chemotherapy in a randomized Phase III trial conducted in France [14].

## Radiation-induced DNA damage and repair

**Fig. 2:**
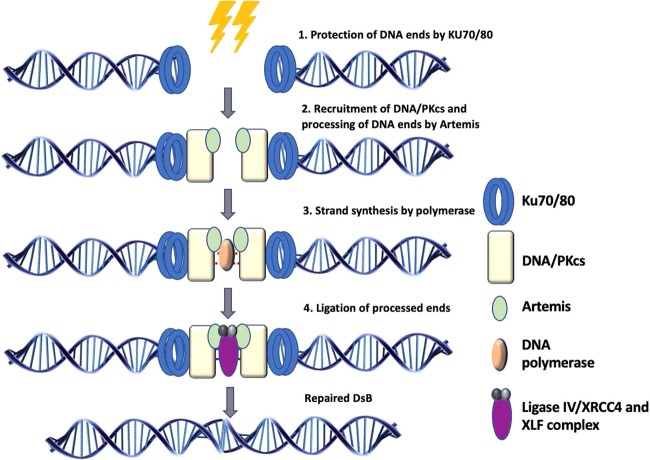
Major steps in NHEJ repair. Broken DNA ends are protected by Ku70/80 before the recruitment of DNA/PKcs and processing by artemis. Damaged bases are replaced and broken ends are ligated.

Upon cellular exposure to radiation, charged particles either directly pass through and ionize DNA or produce reactive oxygen species by ionizing nearby water molecules that in turn react with DNA. These interactions elicit lethal DNA damage. Whereas reactive free radicals can induce a wide gamut of lesions, DNA double-strand breaks (DSBs) are credited to be the most cytotoxic lesions generated from radiation. DSBs involve breaks in the phosphodiester backbone of both strands of DNA separated by ~10 bases or less [15]. The production of DSBs increases linearly with radiation dose [16]. In fact, radiation induces approximately 850 pyrimidine lesions, 450 purine lesions, 1000 single-strand breaks, and on average 30 DSBs/cell/Gy of low linear energy transfer γ-radiation [17, 18]. The standard fractionating dose of 2 Gy/cycle is capable of inducing roughly 3000 DNA lesions per exposed cell, and merely 40 DSBs are required to induce cell death [17, 19]. Generation of free radicals that may lead to DNA damage also occurs during normal cellular metabolic processes, recombination during meiosis, and DNA replication. Evolutionarily, eukaryotes have developed the innate ability to repair DNA. Homologous recombination (HR) and nonhomologous end joining (NHEJ) are the major pathways responsible for repairing DSBs. HR requires homologous DNA sequences from sister chromatids to repair DSBs. HR is therefore restricted to phases of the cell cycle where sister chromatids are available (late S to G2 phases) [20]. In contrast, NHEJ is a promiscuous repair mechanism that directly ligates two broken ends and does not require sequence homology. Hence, NHEJ occurs at all stages of the cell cycle. NHEJ is more error-prone than HR and often includes base deletions and insertions [21]. NHEJ is initiated by Ku proteins. Ku is a heterodimeric protein consisting of Ku70 and Ku80 subunits. It has a high affinity for the ends of DNA and once bound, forms a holoenzyme with DNA-dependent protein kinase catalytic subunit (DNA-PKcs). The DSB termini are processed via activation of artemis, polynucleotide kinase/phosphatase (PNKP), and aprataxin-PNKP-like factor (APLF) if necessary. Subsequently, other proteins involved in the repair pathway get phosphorylated including autophosphorylation of DNA-PKcs. Then gaps are filled by polymerase μ and γ followed by ligation by ligase IV, X-ray repair complementing 4 (XRCC4), and XRCC4-like factor (XLF) (Fig. 2). It should be noted that while Ku is drafted to the site of all DSBs, DNA-PKcs only participates in repair of complex DSBs [22].

## Radiation-induced DNA damage and proliferation

The cell cycle involves a highly regulated set of events that allows cells to proliferate. During the cycle, cells pass through the G_0_ (resting), G_1_ (first growth phase), S (DNA replication), G_2_ (growth), and M (mitosis) phases. Progression through the cell cycle is regulated by cyclin-dependent kinases (CDKs) and cyclins. CDKs remain inactive until they are bound to their associated cyclin subunits. The availability of cyclins is controlled by regulating their synthesis and proteolysis. When cyclins bind CDKs, the conformation of the CDKs is switched to an active state, thereby phosphorylating downstream molecules necessary for cell-cycle progression. Checkpoints are responsible for sensing defects during DNA synthesis and chromosome segregation to maintain cell-cycle integrity. Checkpoint pathways maintain CDKs in inactive states until a DNA lesion is resolved. It is crucial that cells maintain tightly regulated mechanisms to direct cell-cycle progression, especially when cells confront cellular stressors such as radiation. If checkpoints are too tightly regulated, while there would be less occurrences of chromosomal instability, cell proliferation would be significantly attenuated, and survival of the organism would be compromised. In contrast, if checkpoints are bypassed easily, cells would proliferate regardless of the presence of DNA damage and ultimately increase risks of chromosomal instability and cancer development.

Activation of ataxia telangiectasia mutated (ATM) subsequent to recognition of radiation-induced DSBs is a key event in activating checkpoints. Sensing DNA damage in the G1 phase postirradiation results in phosphorylation of H2AX adjacent to the DSB predominantly by ATM [23]. ATM and Chk2 stabilize p53 by dissociating it from MDM2, thereby activating downstream effectors such as p21. Accumulation of p21 in G1 inhibits cyclin/CDK complexes and deters cell-cycle progression. p21 binds to cylin E/CDK2 and cyclin D/CDK4/6 complexes. Deng et al. reported that complete knockout of p53 or p21 results in total loss of the G1 checkpoint [24]. In addition, ATM stabilizes p21 encoding mRNA by activating p38 MAPK [25]. Since p53 activation and effector functions involve transcriptional activation subsequent to posttranscriptional modifications, cell-cycle arrest may require 4–6 h post irradiation. Consequently, cells may progress to the S phase even with high doses of radiation administered in the late G1 phase [26]. DNA damage during the S phase may disturb replication fork progression, and damage to bases may result in point mutations from base mispairings. In addition, formation of DNA–DNA or DNA–protein crosslinks could impede replication fork progression. If DSBs remain unrepaired, further DSBs and chromosomal breaks may occur. Hence, following DSB formation, cell-cycle progression is halted to allow repair. Radiation may also induce cell-cycle arrest in the G2 phase of the cell cycle. The G2/M checkpoint prevents cell division until DSBs are efficiently repaired. Entry into mitosis is induced by the cyclin B1/CDK1 complex. DNA-damaging agents such as radiation disrupt feedback loops that are responsible for further activation of cyclin B1/CDK1. DSBs activate ATM, which phosphorylates Chk2 which phosphorylates Cdc25 resulting in inactivation of cyclin B1/CDK1. The consequence of this chain of events is rapid G2 arrest. The role of p53 in G2/M arrest needs further investigation [27]. There is a caveat to G2/M arrest; increasing evidence suggests that cells are released from G2 arrest after reduction of DSBs below a 10–20 DSB threshold [28]. Regulation of both G1/S and G2/M cell-cycle checkpoints maintain proper cell proliferation.

## Immunomodulatory effects of radiation

In addition to cytotoxic effects, radiation plays a role in immune modulation in the tumor and the tumor microenvironment. Radiation augments antigen-specific antitumor immune responses through various proposed mechanisms. These mechanisms include: (a) increasing activation and proliferation of tumor-infiltrating lymphocytes, (b) altering chemokines that preferentially recruit cytotoxic T lymphocytes and lead to MHC I upregulation, (c) fostering the release of tumor neoantigens via inflammatory cell death, and (d) activation and migration of dendritic cells. Furthermore, an abscopal effect has been noted where irradiation in one area results in tumor regression outside the field of radiation. Interestingly, one of the first reports of this effect was in head and neck cancer in the early 1900s; it is a rare phenomenon [29]. Radiation in combination with immunotherapy has therefore gained traction for HNSCC evidenced by the numerous ongoing clinical trials (Table 1). Keynote 048 showed that pembrolizumab/chemotherapy had a superior overall survival with comparable safety in the overall population versus cetuximab/chemotherapy leading to FDA approval of pembrolizumab as a first-line standard-of-care treatment for recurrent or metastatic HNSCC. Reports of abscopal effects induced by radiation have been noted with higher doses of radiation. For this reason, clinical trials investigating immune checkpoint blockade (ICB) and stereotactic body radiation therapy (SBRT) where precise intense doses can be administered in a shorter period of time as compared with conventional fractionation schemes, are being explored. High ablative doses of RT may superiorly potentiate the abscopal effect compared with fractionated RT [30]. The timing and dose schedules of RT and ICB are still being optimized, but concurrent administration with hypofractionated SBRT seems reasonable.

**Table 1:** Clinical trials combining RT and immune checkpoint blockade for HNSCC.

Study identifier	Disease type	Phase	Design	Primary endpoint
NCT03799445	Locally advanced	II	RT + immune checkpoint inhibition (nivolumab + ipilimumab)	DLTs, CR rate (6 months) and PFS (2 years)
NCT03509012	Locally advanced	I	RT + cisplatin + durvalumab	DLTs (28 days post therapy) and AEs (90 days post therapy)
NCT03426657	Locally advanced	II	RT + durvalumab + tremelimumab	Feasibility, tumor-infiltrating CD8+ T cells and DLTs
NCT0351906	Locally advanced	I/II	RT + cetuximab + durvalumab	PFS (2 years)
NCT02999087	Locally advanced	III	Arm 1: RT + cisplatin	PFS (6 years)
			Arm 2: RT + cisplatin + avelumab	
NCT02764593	Locally advanced	I	Arm 1: Cisplatin + nivolumab	Dose-limiting toxicity (28 days post therapy)
			Arm 2: High dose cisplatin + nivolumab	
			Arm 3: Cetuximab + nivolumab	
			Arm 4: RT + nivolumab	
NCT03623646	Locally advanced	II	Arm 1: RT + cisplatin	Progression (1 year)
			Arm 2: RT + durvalumab	
NCT03546582	Recurrent or second primary	II	Arm 1: SBRT + pembrolizumab	PFS (2 and 5 years)
			Arm 2: SBRT	
NCT03522584	Recurrent or Metastatic	I/II	SBRT + durvalumab + tremelimumab	Safety and AEs (2 years)
NCT03317327	Recurrent or second primary	I/II	RT + nivolumab	AEs (6 months post therapy)
NCT03212469	Metastatic	I/II	SBRT + durvalumab + tremelimumab	DLTs
NCT03085719	Metastatic	II	Arm 1: High dose RT + pembrolizumab	ORR (1 year)
			Arm 2: High dose + low dose RT + pembrolizumab	
NCT02684253	Metastatic	II	Arm 1: Nivolumab	BOR (96 weeks)
			Arm 2: SBRT + nivolumab	
NCT03283605	Metastatic	I/II	RT + immune checkpoint inhibition (durvalumab + tremelimumab)	Acute toxicity (3 months) and PFS (6 months)
NCT03313804	Advanced or metastatic	II	SBRT or fractionated RT + nivolumab or pembrolizumab or atezolizumab	PFS (6 months)

Data are from www.clinicaltrials.gov.

*DLTs* dose-limiting toxicities, *AEs* adverse events, *CR* complete response, *DFS* disease-free survival, *PFS* progression-free survival, *ORR* overall response rate, *BOR* best overall response.

## Mechanism of radiation resistance in HNSCC

Radiation sensitivity is dependent on the amount of DNA damage induced within the cell and the cell’s ability to activate repair mechanisms through DNA-damage response (DDR) pathways [31]. Subsequent to failure of DDR activation and repair of cellular DNA, cells cannot divide, and die via mechanisms that include apoptosis, necrosis, senescence, mitotic catastrophe, or autophagy [32]. Radioresistant cancer cells have an increased propensity to augment the DDR rate. As mentioned earlier, the major radiation-induced DSB repair mechanism is NHEJ. Multiple proteins involved in NHEJ are associated with radioresistance in HNSCC. For example, overexpression of TRIP13 promotes NHEJ repair and treatment resistance in vitro [33]. In addition, Ku80 expression is correlated with radiation resistance in vitro, and abrogating Ku80 restores sensitivity [34]. In fact, Ku80 protein expression is associated with locoregional failure and death, post radiotherapy [35]. ATM is a key player in DSB repair, and its kinase activity is responsible for the activation of key proteins such as CHK2. In addition, ATM is crucial for phosphorylation of DNA-PKcs at Thr-2609 in response to radiation, thereby playing a fundamental role in NHEJ repair [36]. Therefore, not surprisingly, disrupting ATM function permits radiosensitization of tumors [37].

There has been increased emphasis on elucidating the role of HPV16 in response to radiation. While HPV16 accounts for between 60 and 80% of HPV-related HNSCC, HPV18 accounts for ~2.5% and other subtypes (HPV33, 35, 31, 52, 39, and 45) have been reported to account for between 11 and 16% [38]. HPV16 is a DNA virus with oncogenic properties. Many studies have made etiological associations between HPV16 and oropharyngeal squamous cell carcinomas originating from the base of tongue and tonsils, and to a lesser extent in laryngeal and oral cavity cancers [39, 40]. Expression of HPV16 E6 and E7 oncoproteins allows cells to bypass normal antiproliferative control mechanisms and supports tumorigenesis. E6 protein can bind to p53 resulting in ATP-dependent degradation of p53, whereas E7 targets and binds to the retinoblastoma tumor suppressor protein pRb, impairing function. Since HPV16-associated HNSCC is relatively radiosensitive and given the role of HPV16-associated oncoproteins in inactivating wild-type (WT) p53 function, it is possible that failure to respond to radiation in the same subset of patients can be overcome by restoring p53 function through mechanisms such as gene therapy.

## P53 activation/inactivation

A crucial element of the DDR machinery is the activation of tumor suppressor protein p53 by kinases such as ATM and DNA-PKcs. The major outcomes associated with p53 induction are cell-cycle arrest and DNA repair or apoptosis [41]. Tissue type, extent of damage, duration of stress, and the cell’s environment determine these outcomes. Details of this decision-making process are especially relevant in clinical settings where p53 status may be important for response to treatment with DNA-damaging agents such as radiation. Low levels of transient stress could trigger repairable DNA damage; then a survival response is elicited, and p53 acts as a protector. In this case, activation of p53 mediates cell-cycle arrest and DNA repair. In contrast, high levels of sustained stress, which lead to irreparable damage, activate the killer functions of p53 [42]. When the killer function is activated, p53 eliminates damaged cells via apoptosis or cellular senescence [43].

Mutations in TP53 have been associated with high rates of locoregional recurrence, increased resistance to therapy, and reduced survival. TP53 mutations may be classified as disruptive or nondisruptive. Disruptive mutations involve aberrations within the DNA binding domains or a truncated p53 protein due to the presence of an early stop codon; these alterations result in a complete loss of function. In contrast, nondisruptive mutations partially affect the normal functionality of p53 [44]. Cellular exposure to radiation induces DSBs that activate p53. In cancer cells with mutated p53, repair of radiation-induced DNA damage is substandard yet proficient enough to generate clones with an accumulation of genetic mutations that confer resistance. Furthermore, loss-of-function mutations in p53 disable cell-cycle arrest and apoptosis resulting in cell survival and radiation failure [45]. Dominant negative mutations may also inactivate the function of the remaining WT allele. Similarly, gain-of-function mutations may lead to resistance to DNA damage-induced cell death via downregulation of proapoptotic genes and upregulation of prosurvival genes [46]. Gain-of-function mutations were identified as p53 aberrations that conferred additional oncogenic properties to tumor cells. The cell type, stimuli, position, and nature of the substitution of p53 determines its gain-of-function potential [4]. Gain-of-function mutations can also increase DNA repair, induce replicative stress and genomic instability, inactivate ATM, promote proliferation, migration, invasion, and deregulate metabolism, all of which may contribute to resistance [47].

**Fig. 3:**
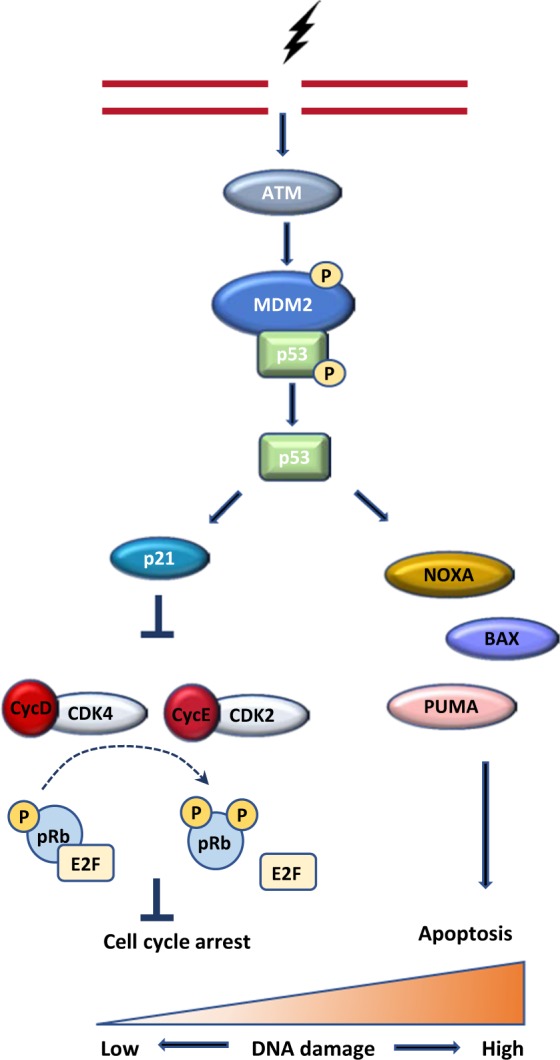
p53 determines cell fate following induction of DNA damage. Activation of ATM activates p53, which depending on severity of the damage executes one of the two functions: cell-cycle arrest or apoptosis. Cells undergo cell-cycle arrest if damage is repairable and apoptose if irreparable.

Induction of ATM by radiation inhibits MDM2, the negative regulator of p53. DNA repair at the G_1_/S and G_2_/M checkpoints prevents propagation of errors to daughter cells. Activated p53 binds to and transactivates the p21 promoter [48]. The predominant consequence of p21 activation is the inhibition of Cdk2 and Cdk4 allowing a hypophosphorylated form of pRb to remain bound to E2F1 resulting in G_1_ arrest (Fig. 3) [49]. E2F1 is a transcription factor with the pivotal function of regulating genes responsible for cell proliferation in a cell-cycle dependent manner.

Although HPV16-positive oropharyngeal HNSCCs are relatively radiosensitive the mechanism is unclear. Speculations include an association with p53 status. TP53 mutations are present in 75–85% non-HPV16-related HNSCC [50]. Most HPV16-positive tumors have WT p53 and are more sensitive to radiation than HPV-negative tumors. Radiation-sensitive cells generally have higher basal levels of p53 mRNA while suppression of p53 in tumors results in increased resistance to radiation. With functional p53, apoptotic pathways can be initiated upon exposure to radiation. In contrast, HPV16 E6 protein ubiquitinates p53, targeting it for degradation. Hence, cell-cycle arrest is inhibited in response to DNA damage and makes cells more susceptible to genomic instability [51]. This may be a mode of escape for radiation-resistant HPV16-associated tumors. The differential response to treatment between HPV16-positive and -negative HNSCC provides a valid reason to consider treatment de-escalation for HPV16-positive patients. However, deeper knowledge of the biology is necessary to justify this approach. Clinical trials have been designed to assess the clinical outcomes associated with reduced radiation treatment in HPV16-driven HNSCC (Table 2).

**Table 2:** De-intensification clinical trials with reduced radiation in HNSCC.

Study identifier	Phase	Design	Primary endpoint
NCT03416153	II	Arm 1: 70 Gy + carboplatin + paclitaxel	LRR (1 year)
		Arm 2: Initially prescribed 70 Gy + carboplatin + paclitaxel followed by reduction to 54 Gy (to high risk PTV) and 43.2 Gy (to low risk PTV)	
NCT03323463	II	30 Gy + cisplatin or (+carboplatin + 5-fluorouracil in cycle 1 or 2)	Comparable with standard chemoradiation (2 years)
NCT01530997	II	54–60 Gy + weekly cisplatin	PCR
NCT01716195	II	Arm 1: Paclitaxel + carboplatin followed by 6 weeks radiation	PFS up to 2 years
		Arm 2: Paclitaxel + carboplatin followed by 5 weeks radiation	
NCT01084083	II	Arm 1: Paclitaxel + cisplatin followed by 27 fractions of IR + cetuximab	24 month PFS
		Arm 2: Paclitaxel + cisplatin followed by 33 fractions of IR + cetuximab	
NCT01088802	II	Dose de-escalation from 70–63 Gy and dose de-escalation from 58.1–50.75 Gy	Grade 3+ late toxicity, QOL and AEs
NCT01706939	III	Arm 1: Standard 70 Gy + carboplatin	PFS at 3 years
		Arm 2: 56 Gy + carboplatin	

Data are from www.clinicaltrials.gov.

*LRR* local regional recurrence, *PCR* pathologic complete response rate, *PFS* progression-free survival, *QOL* quality of life, *AEs* adverse events.

Tumors of lymphoid or myeloid lineage are usually more sensitive to radiation than epithelial-derived tumors like HNSCC, which undergo significantly less apoptosis [52]. The desired outcome for patients treated with radiation is to activate the killer functions of p53. With sufficient DNA damage, p53 induces expression of a number of genes involved in apoptosis. Radiation induces apoptosis by activating caspases via intrinsic or extrinsic pathways. Through the intrinsic pathway, p53 promotes transcription of proapoptotic proteins primarily belonging to the BCl-2 and BH3-only family. Some of these proteins include Bax, Puma, Noxa, and Bim [53, 54]. p53 may also upregulate genes that lower the apoptotic threshold and mediate repression of antiapoptotic genes [55–57]. The extrinsic apoptotic pathway also called the death receptor pathway requires activation of TNF receptor family members by specific ligands. Radiation increases many of these receptors leading to activation of caspases. Several studies in mice have highlighted p53’s ability to induce apoptosis thereby inhibiting spontaneous tumors, delaying tumor progression in mice with activated oncogenes, and inducing regression of fully developed tumors. p53 null mice were reported to be susceptible to spontaneous thymic lymphomas, and apoptosis of thymocytes post irradiation was dependent on p53 [58]. In addition, tumors with mutated T antigen in the p53 binding domain showed rapid growth and reduced apoptosis [59]. This suggests that p53 restricts tumor expansion by inducing apoptosis. Furthermore, restoring normal p53 in hepatocellular carcinomas and sarcomas resulted in tumor regression via p53-mediated senescence [60, 61].

## Epidermal growth factor receptor

EGFR is a tyrosine kinase transmembrane receptor that is activated by ligands including EGF and transforming growth factor (TGFα and β). Ligand-mediated EGFR activation results in autophosphorylation of the intracellular domain with downstream activation of PI3K and Ras pathways to elicit survival and proliferation. EGFR is overexpressed in over 90% of HNSCC and is associated with poor clinical outcome [62]. Patients with high EGFR show higher rates of postradiation locoregional failure, suggesting that EGFR contributes to radiation resistance [63]. Radiation mimics ligand–receptor interaction by inducing autophosphorylation of EGFR [64], which leads to hyperactivation of Ras and PI3K pathways that support proliferation and survival of tumor cells, and ultimately radiation failure. Another caveat of radiation is that after autophosphorylation and Ras activation, the downstream effector MAPK mediates synthesis of EGF, amphiregulin, and TGF monomers that induce an autocrine loop, leading to overactive proliferative pathways.

EGFR may modulate repair of radiation-induced DSBs via formation of an EGFR–DNA-PK complex [65]. There is uncertainty about the intracellular site of the initial EGFR and DNA-PK interaction. These proteins may associate in the nucleus following translocation of EGFR, which results in increased repair of radiation-induced DNA damage [66]. The EGFR–DNA-PK complex is formed within 5 min of radiation with optimal activity of DNA-PK at ~10 min [65, 67]. EGFR is present on the plasma membrane, and upon radiation, evades internalization and degradation, and translocates to the nucleus [68]. In addition, EGFR in the perinuclear space translocates into the nucleoplasm in a ligand-independent manner when subjected to radiation [65]. DNA-PKcs and Ku are generally nuclear proteins, but may localize in lipid rafts and interact with membrane proteins, and can translocate from the nucleus to cytosol [69, 70]. Regardless of where the interaction occurs, it undoubtedly constitutes a critical component of EGFR-mediated radioprotection.

Cells with WT EGFR display diminished radiosensitivity compared with mutant counterparts. However, the radioprotective effect of WT EGFR is lost in DNA-PKcs-deficient cells [66, 71]. EGFR also regulates transcription of ATM. ATM plays a pivotal role in phosphorylation of DNA-PKcs, which mediates DSB repair [36]. When cancer cells are irradiated and undergo cell-cycle arrest, EGFR contributes to successful repair, allowing cells to exit the arrested phase.

For these reasons, EGFR inhibition and radiation has been an attractive treatment combination. Clinical trial data suggested that cetuximab plus radiation was superior to radiation alone for patients with locoregional disease [72]. However, the RTOG 0522 study demonstrated no benefit of cetuximab-radiation over cisplatin-radiation for treatment of locoregionally advanced oropharyngeal HNSCC [73]. Furthermore, in two randomized trials of definitive chemoradiation with concurrent cisplatin versus cetuximab, cetuximab resulted in inferior outcomes to platinum with similar overall toxicity [74, 75]. RTOG 1016 reaffirmed concurrent cisplatin as the standard of care for platinum eligible patients. Cetuximab (versus altered fractionated RT alone) is an option for nonplatinum eligible patients, both HPV16-positive and HPV16-negative. To this end, NRG HN 004 is currently testing RT/cetuximab versus RT/anti-PD1 in noncisplatin eligible patients. Results of these trials highlight the need to predict patient populations who will respond to the combination treatment and/or identify other targetable molecular markers that may improve patient outcome. The future of EGFR inhibition in HNSCC likely depends on predictive biomarkers.

## Additional players in radiation resistance

Phenotypic and functional heterogeneity of HNSCC contributes to the challenges faced when treating the disease. Genomic instability results in an accumulation of mutations that contribute to tumor heterogeneity and developmental diversity of cells within the tumor mass. CSCs have been identified within tumors as self-renewing cells with the potential to differentiate into heterogenous lineages in the tumor. Although these cells account for only 1–5% of the HNSCC population, they have been implicated in radiation resistance, recurrence, and metastases. The mechanism of CSC-mediated radioresistance is unclear, but these slow cycling cells are believed to exhibit increased efficiency in DNA repair (via regulation of DNA repair genes and activation of DNA-damage checkpoint responses) and an upregulation of antiapoptotic proteins. Paradoxically, there are reports highlighting that radiation itself may induce non-CSCs to become CSCs [76].

## Radiation sensitizers in HNSCC: targeting DNA repair pathways

Since radiation-induced DSBs are primarily repaired via NHEJ, targeting NHEJ has the potential to radiosensitize tumor cells. NHEJ repair consists of four stages including termini recognition, bridging, processing, and ligation of DNA. In the initial step, the Ku70/Ku80 complex is recruited to the DNA terminus of a DSB, and then recruits DNA-PKcs leading to formation of a heterotrimeric complex. Ku localizes within seconds of DSB formation due to high affinity for DNA termini and high concentration within the cell. There are ~0.5 × 10^6^ Ku molecules per cell with a DNA binding affinity between 2.4 × 10^−9^ and 5 × 10^−10^ M^−1^ [77, 78]. Ku forms a ring structure and slides onto the DNA ends where it binds to the sugar backbone of the DNA rather than to the bases [79]. Binding of Ku protects DNA ends from nonspecific processing thereby maintaining DNA stability. When Ku-deficient cells are irradiated in S phase, severe chromosome instability is observed [80]. Therefore, inhibition of Ku proteins with concurrent radiation offers an attractive treatment option in cancer. Li et al. showed that depletion of Ku70 or Ku80 sensitizes pancreatic cancer cells to radiation [81]. DNA-PK is responsible for phosphorylation of other key proteins needed in subsequent steps of the repair pathway and without it, repair would be compromised. Therefore, several DNA-PK inhibitors have been designed, including wortmannin and LY294002. Wortmannin is a potent inhibitor of DNA-PK by making an irreversible covalent modification on Lys802 in the active site of DNA-PK, which is crucial for a necessary phosphate transfer reaction. This compound is a general noncompetitive inhibitor of PI-3 kinases. LY294002 on the other hand is a nonspecific competitive inhibitor that binds irreversibly to the DNA-PK kinase domain [82]. Both these compounds have failed to make it into the clinic due to lack of specificity, poor solubility, and severe toxicity [83]. NU7026 and NU7441, more selective DNA-PK inhibitors, have shown efficacy in vitro but failed to make a clinical impact. One of the major issues with NU7026 was the metabolic instability of the morpholino ring. This challenge made it difficult to deliver appropriate concentrations of the drug in the time frame required for synergy with radiation [84]. More recently, a selective inhibitor of NHEJ, VX-984, that preferentially affects transformed cells was described [85]. Studies in glioblastoma cell lines and tumor-bearing mice showed that VX-984 enhances radiosensitivity of brain tumors [86]. Notably, VX-984 dose dependently inhibited radiation-induced DNA-PKcs phosphorylation and attenuated clonogenic survival [86]. The safety and tolerability of VXλ84 in combination with chemotherapy was assessed in participants with solid tumors in clinical trial NCT02644278. The study was discontinued due to business-related reasons limiting the conclusions [87].

Additional attempts have been made to target DNA end-processing to disrupt NHEJ repair subsequent to radiation-induced DSBs. DNA ends are processed to facilitate ligation since very rarely are blunt ends formed during radiation damage [88]. DSB ends may contain 5′ hydroxyls or 3′ phosphates that cannot be ligated. PNKP phosphorylates the 5′ hydroxyl termini and dephosphorylates the 3′ phosphate termini that often result from radiation treatment [89]. A12B4C3, a noncompetitive inhibitor, has been designed to dysregulate PNKP’s phosphatase activity by disrupting protein secondary structure and leading to radiation sensitivity. The inhibitor sensitizes breast and lung cancer to radiation in vitro and needs further assessment in vivo [90]. Artemis is another protein involved in processing the DNA ends in preparation for repair. Phosphorylation of artemis by DNA-PK permits its endonuclease activity, which nicks 5’ overhangs on a duplex end and displays its hairpin-opening activity [91]. Likewise, artemis has single-strand specific 5′-3′ exonuclease activity. Optimizing a drug to inhibit artemis may show efficacy in treating cancer.

The final step of NHEJ repair is ligation of broken ends and disintegration of the NHEJ complex. XRCC4 stabilizes DNA ligase IV, enhancing its activity by promoting adenylation, which ligates discordant DNA ends across gaps [92, 93]. While phosphorylation of XRCC4 disrupts its ability to bind DNA, it maintains the capacity to bind DNA ligase IV [94]. Therefore, phosphorylation of XRCC4 may dissociate the XRCC4/ligase IV complex from DNA after repair. Additional proteins including XLF and APLF stimulate ligation by the XRCC4–DNA ligase complex [95, 96]. XLF encourages ligation of mismatched and noncohesive DNA ends [95].

There is uncertainty about whether DNA-PKcs is released from the repair site before ligation. DNA-PKcs is released from DNA after conformational changes resulting from autophosphorylation events [97]. However, Ku may remain on the DNA molecule after ligation until it is ubiquitylated and degraded [98]. Inhibitors of DNA ligase IV have been developed to inhibit the ligation step of NHEJ repair. L189 displayed very poor specificity; it could inhibit all three mammalian ligases [99]. SCR7 was a more specific inhibitor of ligase IV. Treatment with SCR7 resulted in accumulation of DSBs and subsequent activation of apoptotic pathways in vitro and in vivo. In fact, tumor-bearing mice treated with SCR7 and radiation displayed significant reduction in tumor burden [100]. Further investigation of specific NHEJ inhibitors is necessary. Treatment with a NHEJ inhibitor has the potential to substantially improve radiosensitivity (Table 3).

**Table 3:** NHEJ proteins and molecular inhibitors.

NHEJ repair protein	Inhibitor	References
DNA- PKcs	Wortmannin	Davidson et al. [82]
	NU7026	Nutley et al. [84]
	NU7441	Timme et al. [86]
	LY294002	Davidson et al. [82]
	VX-984	Khan et al. [85]
PNKP	A12B4C3	Freschauf et al. [90]
Ligase IV	L189	Chen et al. [99]
	SCR7	Srivastava et al. [100]

## Conclusion

Radiation is a potentially curative treatment for HNSCC if used within the appropriate patient population. Radiation induces significant DSBs, which if unrepaired, lead to cell death. Unfortunately, cancer cells have cleverly devised mechanisms to repair radiation-induced DNA damage predominantly by NHEJ. Identifying new targetable players in NHEJ repair could lead to combination therapies to improve disease control and patient survival. With knowledge of p53 and EGFR involvement in the repair process, identification of binding partners may highlight additional avenues to target in order to improve response. Moreover, combination approaches with immunotherapy may be efficacious.
